# Development and validation of a haplotype‐free technique for non‐invasive prenatal diagnosis of spinal muscular atrophy

**DOI:** 10.1002/jcla.23046

**Published:** 2019-09-25

**Authors:** Xianda Wei, Weigang Lv, Hu Tan, Desheng Liang, Lingqian Wu

**Affiliations:** ^1^ Center for Medical Genetics School of Life Sciences Central South University Changsha China; ^2^ Hunan Jiahui Genetics Hospital Changsha China

**Keywords:** cell‐free DNA, digital PCR, non‐invasive prenatal diagnosis, prenatal screening, spinal muscular atrophy

## Abstract

**Objective:**

To develop a technique for non‐invasive prenatal diagnosis of spinal muscular atrophy and validate its performance.

**Study Design:**

Pregnant women with 1 copy of *SMN1* and male fetuses were enrolled. Seventeen women were included in test set A, and 10 of them were selected into test set B randomly and blinded. The two sets were tested independently by two different researchers blinded to fetal genotypes. Fetal DNA fractions were calculated based on the relative proportion of mapped chromosome Y sequencing reads. An algorithm was developed to decide fetal *SMN1* copy numbers.

**Results:**

The concordance rate with the results of MLPA testing of amniocyte DNA was 94.12% in test set A and 90% in set B. For all tests with a classifiable result, the percent of agreement with the results of MLPA testing of amniocyte DNA was up to 100% (25/25).

**Conclusion:**

We have developed a direct, rapid, and low‐cost technique, which has a potential to be utilized for first‐trimester non‐invasive prenatal diagnosis and screening for spinal muscular atrophy with considerable reliability and feasibility.

## 1 INTRODUCTION

Spinal muscular atrophy (SMA) is one of the most common autosomal recessive diseases causing infant mortality, with an incidence around 1 in 10 000 births.^1^ About 81%~95% of SMA patients have no detectable exon 7 of the *SMN1* gene,^2^ which is located in a 1.5‐Mb reverse‐duplicated region containing multiple copies of homologous sequences.^3^ Survival motor neuron 2 (*SMN2*) gene is also located in the 5q13 region, the coding sequence of which differs from *SMN1* only by the 6th nucleotide of exon 7, where a C‐to‐T transition leads to the alternatively spliced isoform translating the non‐functional SMN△7 protein.^4^, ^5^ Prenatal diagnosis is an essential prevention for SMA. Conventional procedure involves invasive approaches for fetal genetic materials such as amniocentesis and chorionic villus sampling (CVS), which harbor risks for miscarriage and infection.^6^ Non‐invasive prenatal diagnosis of SMA in earlier pregnancy would be timely and safer.

The discovery of cell‐free DNA (cfDNA) in maternal plasma has enhanced the development of non‐invasive prenatal testing (NIPT).^7^ Although NIPT for fetal aneuploidies has already been clinically applied, non‐invasive prenatal diagnosis for many single‐gene disorders remains on the developing stage. For NIPD of SMA, a technique by targeted sequencing of cfDNA in maternal plasma and relative haplotype dosage (RHDO) analysis has been previously published.^8^, ^9^ However, this haplotype‐based strategy has several limitations. Firstly, there is a rigid demand for DNA of the probands and parents, as well as adequate informative genomic markers beside the *SMN1* gene.^10^ This limitation restricts the scope of subjects applicable to this test. Secondly, a recombination event may result in incorrect fetal genotype classification if it occurs as a genomic location near the mutation. Thirdly, for de novo *SMN1* mutations with a rate that is reported to be high,^11^ and for germline mosaicism, haplotyping would fail or come out with false‐negative results.

Droplet digital PCR is a technology with high sensitivity, specificity, and accuracy to detect and analyze low‐abundance nucleic acids. Its high resolution is guaranteed by millions of oil droplets generated per test. Utilizing digital PCR, the feasibility of non‐invasive prenatal diagnosis (NIPD) for fetal monogenic disorders has been proved in several studies analyzing cfDNA.^12^, ^13^, ^14^, ^15^, ^16^ In particular, for maternally inherited single nucleotide mutations, the relative mutation dosage (RMD) analysis based on the sequential probability ratio test (SPRT) has enabled detection of a slight increase in the load of the mutant allele in the maternal plasma of heterozygous carriers.^17^ Digital PCR provides an ideal platform for the development of a haplotype‐free test strategy for SMA‐NIPD.

Unlike most other single‐gene disorders, SMA harbors need and potential for a specific design of NIPD technique. The prominent hot spot mutation in the *SMN1* gene, which is the loss of exon 7 copies but not point mutations, implies the utilization of single‐base targeting strategy but disables the application of regular RMD algorithm. The pseudogene *SMN2* that disturbs quantification of *SMN1* proposes a major obstacle. In this article, we present a novel technique that directly analyses *SMN1* gene dosage using droplet digital PCR, as well as the results of performance validation.

## 2 MATERIALS AND METHODS

### 2.1 Design of probes and primers

TaqMan MGB probes were designed at the 6th nucleotide in exon 7 of *SMN1*/*SMN2* gene and intron 3 of the reference *ALB* gene and synthesized by Thermo Fisher Scientific. We designed the length of *SMN1/SMN2* and *ALB* amplicons as short as 75 bp and 72 bp to reduce the bias caused by unbalanced PCR amplification in favor of fetal cfDNA, which is generally shorter than maternal cfDNA.^18^ Sequences of the probes and primers are listed in the Appendix S1. Quantitative PCR was conducted for samples with various *SMN1/SMN2* copy numbers to validate the specificity of the probes.

### 2.2 Droplet digital PCR

RainDrop droplet digital PCR should be performed following standard protocols, through processes including PCR mixture preparation, droplet sourcing, PCRs, and signal sensing. For each PCR, droplets with positive signal for *SMN1/ALB* should be counted using RainDrop Analyst II software.

### 2.3 The digital relative *SMN1* dosage method

Statistical analysis is essential for the determination of *SMN1* copy number from digital PCR data. Based on the principle of Poisson distribution and hypothesis testing, we set up an algorithm called digital relative *SMN1* dosage, as specified and illustrated in the Appendix S1. In short, a hypothesis that fetal *SMN1* copy number equals 1 is established at first. Next, Pr(observed) value is generated for each test of one sample (one data set), which is determined solely by the number of reaction‐positive droplets. Then, through comparing Pr(observed) to the upper and lower thresholds (derived from the number of reaction‐positive droplets and FF) under a certain threshold likelihood ratio (a marker of statistical significance with a default value 2, a higher value represents higher reliability), the algorithm would return one of the three possible outcomes: accept the hypothesis (fetal *SMN1* copy number = 1)/reject the hypothesis (fetal *SMN1* copy number = 0 when n*_SMN1_*_/_n*_ALB_* < 0.5; fetal *SMN1* copy number = 2 when n*_SMN1_*_/_n*_ALB_* > 0.5)/an unclassifiable result.

### 2.4 Validation of the technique performance

#### 2.4.1 Participants and sample processing

For the validation, we recruited pregnant women seeking SMA prenatal diagnosis on 16 ~ 22 weeks of gestation for this study from the Hunan Jiahui Genetics Hospital and signed informed consent. All of the pregnancies had undergone non‐invasive prenatal screening for fetal aneuploidies by next‐generation sequencing (NGS). Approval was obtained from the Ethics Committee of The Center for Medical Genetics, School of Life Sciences, Central South University, Hunan, China. For each participant, 6 ~ 10 mL of peripheral blood was collected in BCT Cell‐Free DNA Blood Collection Tube (Streck) and 10 mL of blood was collected into k3‐EDTA acid tubes. Weeks of gestation when sampling blood are listed in Table 1. Plasma was separated after double centrifugation within 6 hours after collection, one at 1600 g for 10 minutes and the second at 16000 g for 10 minutes. We extracted cell‐free DNA from maternal plasma using the QIAamp Circulating Nucleic Acid Kit (Qiagen) following the manufacturer's instructions. The concentrations of cfDNA samples were tested on Qubit (Thermo Fisher Scientific). Amniotic fluid was obtained by amniocentesis, from which fetal genomic DNA was extracted by the phenol‐chloroform method.

**Table 1:** Week of gestation, fetal *SMN1* genotype, fetal DNA fraction, and cfDNA concentration of the 17 samples

Sample number	Week of gestation when sampling	Fetal copy number of *SMN1*	cfDNA concentration (ng/μL)	cfDNA total amount (ng)	Fetal DNA fraction
G3313	21^+6^	1	0.380	13.23	13.97%
G3507	16^+3^	1	0.486	19.44	6.58%
G3515	16^+1^	1	0.745	29.80	8.40%
G3562	18^+4^	2	0.340	10.26	13.12%
G3567	19^+2^	1	0.840	31.01	15.83%
G3612	17^+5^	0	0.512	20.48	7.27%
G3673	16^+3^	0	0.860	34.40	10.22%
G3731	17^+2^	0	0.800	28.14	9.29%
G3736	18^+2^	0	0.400	13.86	12.83%
G3780	21^+3^	2	0.400	11.94	10.55%
G3846	19^+5^	1	0.349	13.96	14.66%
G3854	20^+4^	1	0.408	16.32	12.01%
G3978	18^+6^	1	0.220	8.29	11.79%
G4007	17^+1^	2	0.660	24.49	7.36%
G4032	20^+3^	0	0.210	7.21	16.21%
G4185	19^+1^	1	0.650	22.89	11.48%
G4223	18^+5^	2	0.410	14.28	9.41%

#### 2.4.2 Establishment of test sets

*SMN1/SMN2* copy numbers of all participants and fetuses were quantified by the multiplex ligation‐dependent probe amplification (MLPA) analysis using SALSA MLPA Kit (P060 MRC Holland) according to the manufacturer's instructions. Fetal sex was determined by amplifying the *SRY* gene in amniocyte DNA.

We established test set A of cfDNA from 17 women with 1 copy of *SMN1* (SMA carriers) and pregnant with male fetuses. A researcher blind to fetal genotypes established test set B by randomly selecting 10 samples in the test set A. The other two researchers conducted digital PCR and data analysis for set A and set B independently and blinded to fetal genotypes. In addition, six genomic DNA samples with different *SMN1/SMN2* copy numbers were also included in this study to validate probe specificity.

#### 2.4.3 Determination of fetal DNA fraction

We determined fetal DNA fraction (FF) of the samples based on the relative proportion of mapped chromosome Y (ChrY) sequencing reads, which is the golden standard method for FF determination.^19^ In brief, low‐coverage (0.1×) whole‐genome sequencing was performed for the cfDNA samples. FF in maternal plasma was calculated by comparing the sequence tag density of ChrY in maternal plasma with the sequence tag density of ChrY in male plasma.

## 3 RESULTS

### 3.1 Validation of probe specificity for *SMN1* by quantitative real‐time PCR

Results of TaqMan quantitative real‐time PCR conducted on genomic DNA samples with various *SMN1/SMN2* copy numbers were completely in accordance with the MLPA results. Samples with one or more *SMN2* copies and no *SMN1* copy did not produce fluorescence signal (FAM) of *SMN1*, which proved reliable specificity of the designed TaqMan MGB probes.

### 3.2 Validation of performance

Fetal fraction determined by low‐coverage (0.1×) whole‐genome sequencing ranged from 6.58% to 16.21%, with an average of 11.27% (Table 1). For samples in test set A, 16 had a classifiable *SMN1* copy number, while one sample had an unclassifiable result (Table 2). The concordance rate with the results of MLPA testing of amniocyte DNA in test set A was 94.12% (16/17). For samples in test set B, nine had a classifiable *SMN1* copy number, while one sample had an unclassifiable result (Table 3). The concordance rate with the results of MLPA testing of amniocyte DNA in test set B was 90% (9/10). For all tests with a classifiable result, the percent of agreement with the results of MLPA testing of amniocyte DNA was up to 100% (25/25). The results showed considerable accuracy and precision of the technique to test fetal *SMN1* copy number in cfDNA.

**Table 2:** Results of cfDNA samples in test set A

Sample number	Number of droplets produced	Number of droplets positive for *SMN1* (n*_SMN1_*)	Number of droplets positive for *ALB* (n*_ALB_*)	n*_SMN1_*/n*_ALB_* ^†^	Pr(observed)^‡^	Upper threshold	Lower threshold	Fetal *SMN1* copy number by cell‐free DNA	Fetal *SMN1* copy number by amniocyte DNA
G3313	7 509 922	558	1172	0.501706485	0.665909091	0.660974755	0.642899527	1	1
G3507	7 589 437	641	1287	0.498057498	0.667531120	0.679454682	0.668897741	1	1
G3515	7 476 559	919	1840	0.499456522	0.666908300	0.684909300	0.667741972	1	1
G3562	7 214 377	508	976	0.520491803	0.657681941	0.656580450	0.649001031	2	2
G3567	6 510 824	1219	2452	0.497145188	0.667937892	0.688729633	0.682159827	1	1
G3612	7 093 361	431	1024	0.420898438	0.703780069	0.693910900	0.656060557	0	0
G3673	7 068 369	900	2107	0.427147603	0.700698370	0.684916625	0.672097403	0	0
G3731	7 406 514	1339	2905	0.460929432	0.684495759	0.682411499	0.672372092	0	0
G3736	7 903 682	545	1328	0.410391566	0.709022958	0.689770750	0.673605329	0	0
G3780	4 182 515	526	897	0.586399108	0.630358398	0.669983619	0.640830268	2	2
G3846	7 288 518	305	613	0.497553018	0.667755991	0.698248252	0.669674650	1	1
G3854	6 577 602	480	926	0.518358531	0.658605974	0.658261929	0.649566182	1	1
G3978	7 811 029	266	528	0.503787879	0.664987406	0.661969747	0.646302039	1	1
G4007	7 611 593	1124	2052	0.547758285	0.646095718	0.667949223	0.649494269	2	2
G4032	4 607 289	189	457	0.41356674	0.707430341	0.704116199	0.667720307	0	0
G4185	7 294 012	1141	2314	0.493085566	0.66975398	0.684968443	0.675104247	1	1
G4223	7 198 643	706	1345	0.524907063	0.655777669	NA	NA	unclassifiable	2

Note

^†^ n*_SMN1_*/n*_ALB_*: It is the only index determining hypothesis testing H1. H1: fetal *SMN1* copy number = 0 (in cases that n*_SMN1_*/n*_ALB_* < 0.5) or fetal *SMN1* copy number = 2 (in cases that n*_SMN1_*/n*_ALB_* > 0.5).

^‡^ Pr(observed): Pr(observed) = n*_ALB_*/(n*_ALB_* + n*_SMN1_*). It is a value entirely depending on the data of one single test on one sample. Fetal *SMN1* copy number is determined by comparing the value of Pr(observed) with the upper/lower thresholds. Find details in the Appendix S1.

**Table 3:** Results of cfDNA samples in test set B

Sample number	Number of droplets produced	Number of droplets positive for *SMN1* (n*_SMN1_*)	Number of droplets positive for *ALB* (n*_ALB_*)	n*_SMN1_*/n*_ALB_* ^†^	Pr(observed) ^‡^	Upper threshold	Lower threshold	Fetal *SMN1* copy number by cell‐free DNA	Fetal *SMN1* copy number by amniocyte DNA
G3507	6 288 548	440	882	0.498866213	0.667170953	NA	NA	unclassifiable	1
G3515	7 220 904	745	1501	0.496335776	0.668299199	0.679839664	0.672809351	1	1
G3562	5 588 717	556	941	0.590860786	0.628590514	0.664062616	0.641520503	2	2
G3612	7 479 914	385	908	0.424008811	0.702242846	0.696283100	0.653687012	0	0
G3673	6 958 213	657	1562	0.420614597	0.703920685	0.687192209	0.669817422	0	0
G3731	6 295 878	1100	2532	0.434439179	0.697136564	0.683257462	0.671526720	0	0
G3846	7 289 053	279	551	0.506352087	0.663855422	0.645140279	0.657350939	1	1
G3854	6 813 333	321	636	0.504716981	0.664576803	0.660300065	0.647525672	1	1
G4007	6 527 871	936	1772	0.528216704	0.654357459	0.662329211	0.655114379	2	2
G4223	5 705 388	670	1155	0.58008658	0.632876712	0.669258109	0.643905623	2	2

Note

^†^ n*_SMN1_*/n*_ALB_*: It is the only index determining hypothesis testing H1. H1: fetal *SMN1* copy number = 0 (in cases that n*_SMN1_*/n*_ALB_* < 0.5) or fetal *SMN1* copy number = 2 (in cases that n*_SMN1_*/n*_ALB_* > 0.5).

^‡^ Pr(observed): Pr(observed) = n*_ALB_*/(n*_ALB_* + n*_SMN1_*). It is a value entirely depending on the data of one single test on one sample. Fetal *SMN1* copy number is determined by comparing the value of Pr(observed) with the upper/lower thresholds. Find details in the Appendix S1.

## 4 DISCUSSION

A novel NIPD technique has been developed for SMA based on a distinct strategy, with probes and cfDNA‐fit primers designed directly targeting the 6th base of exon 7 in the *SMN1* gene of the fetus. It can detect the loss of *SMN1* exon 7 copy caused by either deletion of DNA fragment containing *SMN1* exon 7 or *SMN1*‐to‐*SMN2* gene conversion. It could address the problems encountered by the haplotype‐based methods. In other words, this technique would be applicable to SMA families without available patient samples or in the conditions that de novo mutations/germline mosaicism/a recombination near the mutation occurred. The validation results exhibited a considerable accuracy and repeatability of the technique. If shown to be robust in future systematically evaluation in a larger population, it may be a safer and more preferable alternative to traditional invasive prenatal diagnosis for SMA families, with an ability to identify affected fetuses at an earlier gestational age.

On the other hand, the feasibility and adaptability of the technique had also been proved by the tests. In terms of the cost, one run with eight samples on the RainDance platform only requires consumable items priced about $600 (including source chip, sense chip, and carrier oil, and would be even lower on a digital PCR platform other than RainDance) and a total experiment time about 6 hours. Besides, in the present study, it is a cost‐saving way to determine fetal fraction by analyzing the existing NGS data of prenatal screening for fetal aneuploidy, which has been generally used as the first‐tier screening assay in clinical practice.

The concentration of cell‐free DNA in maternal plasma and the fetal fraction are key factors influencing the test performance. The samples G3507 and G4223 had classifiable results in one set and unclassifiable in the other set, of which the fetal fraction and the cfDNA concentration are both below average. Quality control standards regarding the two parameters could be established if test data in a larger population will be accumulated in the future. The application of cfDNA‐enrichment techniques to make increased cfDNA concentration and/or fetal fraction may improve the performance and adaptability of the test.

The technique's current version has several limitations. Firstly, the unclassifiable results were from two samples with fetal fractions as 6.58% and 9.41%, possibly indicating a low tolerance to relatively low fetal fractions. Such a proportion of unclassifiable results may lead to excess repeats. Secondly, only male fetuses can be tested because of the chromosome Y method for FF determination. Nonetheless, this is not expected to be an overwhelming obstacle, since alternative FF determination methods for male & female fetuses have already been published.^20^ Thirdly, as this technique quantifies fetal *SMN1* copy number by targeting the 6th base of exon 7, subtle mutations in *SMN1* gene other than loss of exon 7 copy are outside of the test's scope. Fourthly, the technique's performance has not been investigated on multiple pregnancies with or without vanishing fetuses.

## 5 CONCLUSION

We have developed a direct, rapid, and low‐cost NIPD technique for SMA and validated its reliability and feasibility. It has the potential to be utilized for first‐trimester prenatal diagnosis in affected families, as well as prenatal screening in high‐risk population alternative to carrier screening. It can work under several conditions limiting the haplotype‐based NIPD strategy. The technique could provide a practical supplementary to the current prevention system for SMA.

## CONFLICT OF INTEREST

The authors declare no conflict of interest.
