# Supplementary material for: Development and validation of a haplotype‐free technique for non‐invasive prenatal diagnosis of spinal muscular atrophy
Source: J Clin Lab Anal. 2019 Sep 25;34(2):e23046. doi: 10.1002/jcla.23046 (PMC7031576; doi:10.1002/jcla.23046)
Supplement: Supplementary file 1 [file JCLA-34-e23046-s001.docx]

**Supplementary Material**

**Detailed description for the digital relative *SMN1* dosage method**

The digital relative *SMN1* dosage method was constructed based on the principle of Poisson distribution and sequential probability ratio test (SPRT).

Define the target (FAM for *SMN1* or VIC for *ALB*) with the higher number of signal-positive droplets as the major target, and define the other target as the minor target. Define Pr as the proportion of the number of droplets positive only for the major target among the number of droplets positive only for either two targets. Pr(observed) would be determined by data of a real dPCR reaction, while Pr(expected) would be deduced based on the hypothesis that the fetal *SMN1* copy number is not 1 (0 or 2). This theoretical value is determined by the number of droplets positive for the two targets in a real experiment and fetal cfDNA fractions (FF%).

By default, maternal copy number of *SMN1* is 1 and that of *ALB* is 2. Thus, in SMA-NIPD dPCR results of a cfDNA sample, the major target would be *ALB* and the minor target would be *SMN1*.

For one dPCR reaction, the total number of droplets produced is presented with “D”, and the number of droplets positive for *ALB* and *SMN1*is presented with “n*_ALB_*” and “n*_SMN1_*”, respectively.

Pr(observed) = n*_ALB_*/(n*_ALB_*+n*_SMN1_*)

The probability of that no *ALB* segment presents in a single droplet:

P(*ALB*=0) =(D -n*_ALB_*)/D

The probability of that one or more ALB segments presents :

P(*ALB*>0) = 1- P(*ALB*=0)

Define m*_ALB_* as the expected number of *ALB* -positive droplets per droplet, which is the parameter λ for Poisson distribution:

m*_ALB_*=-ln(P(*ALB*=0))

Define m*_SMN1_* as the expected number of *SMN1*-positive droplets per droplet in the hypothesis that fetal *SMN1* copy number is not 1. For fetal *SMN1* copy number=0 (in cases that n*_SMN1_*_/_ n*_ALB_*<0.5),m*_SMN1_* =[1×(1-FF%)+0×FF%] m*_ALB_*/ 2; for fetal *SMN1* copy number=2 (in cases that n*_SMN1_*_/_ n*_ALB_*>0.5),m*_SMN1_* =[1×(1-FF%)+2×FF%] m*_ALB_*/ 2.

The probability of that no *SMN1* presents in a single droplet:

P(*SMN1*=0) = e^-m^*^SMN1^*;

P(*SMN1*>0) = 1- P(*SMN1*=0);

P(*ALB*>0and *SMN1*>0)= P(*ALB*>0) × P(*SMN1*>0);

P(*ALB*>0and *SMN1*=0)= P(*ALB*>0) - P(*ALB*>0and *SMN1*>0);

P(*SMN1*>0and *ALB*=0)= P(*SMN1*>0) - P(*ALB*>0and *SMN1*>0);

Pr(expected) = P(*ALB*>0and *SMN1*=0)/ [P(*ALB*>0and *SMN1*=0)+ P(*SMN1*>0and *ALB*=0)];

Perform hypothesis testing:

H_0_：fetal *SMN1* copy number=1; H_1_: fetal *SMN1*copy number=0(in cases that n_SMN1/_ n_ALB_<0.5) or fetal *SMN1*copy number=2(in cases that n*_SMN1_*_/_ n*_ALB_*>0.5);

Set threshold likelihood ratio at the default value 2.

Define Q_0_ as the theoretical proportion of the number of droplets positive only for *ALB* among the number of droplets positive only for either *ALB* or *SMN1* under the hypothesis H_0_, which equals to2/3;

Define Q_1_ as the theoretical proportion of the number of droplets positive onlyfor *ALB* among the number of droplets positive only for either *ALB* or *SMN1* under the hypothesis H_0_, which is exactly the definition of Pr(expected).

In cases that n*_SMN1_*_/_ n*_ALB_*<0.5:

Upper threshold=[(ln2)/N – ln d]/lng;

Lower threshold=[(ln1/2)/N – ln d]/lng;

In cases that n*_SMN1_*_/_ n*_ALB_*>0.5:

Upper threshold =[(ln1/2)/N – ln d]/lng;

Lower threshold =[(ln2)/N – ln d]/lng;

In which:

d=(1- Q_1_)/(1- Q_0_);

g = [Q_1_ (1- Q_0_)] / [Q_0_ (1- Q_1_)].

**The algorithm to decide fetal copy number of the *SMN1* gene using the digital relative *SMN1* dosage method**


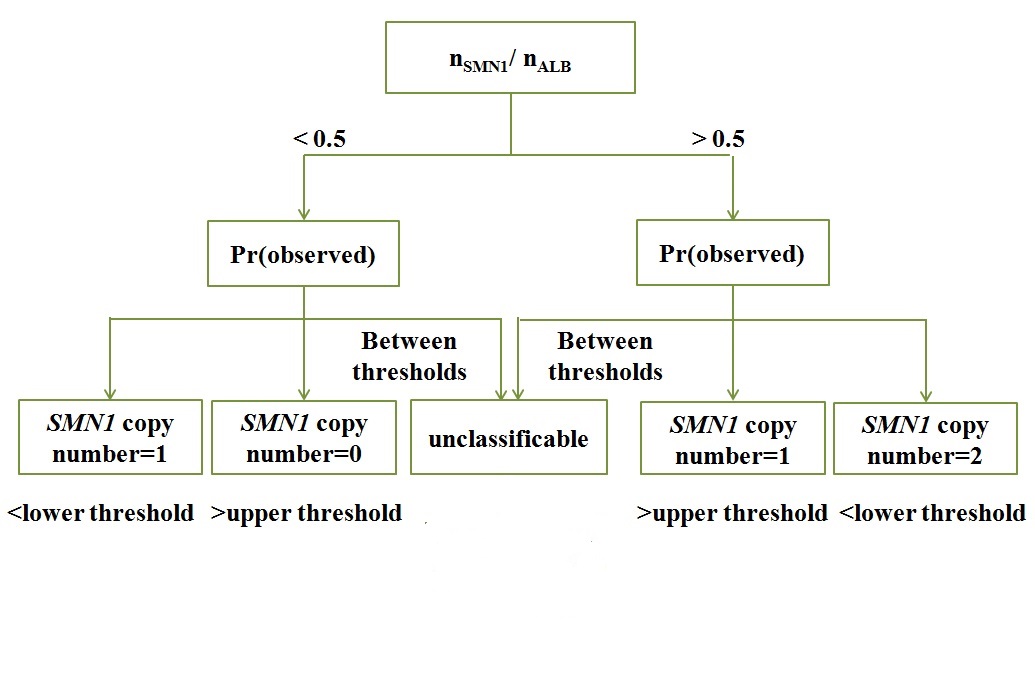


**Sequences of probes and primers (5’-3’)**

**Probes**

*SMN1*：FAM-CAGGGTTTCAGACAAA-MGBNFQ

*SMN2*：FAM-TGATTTTGTCTAAAACCC-MGBNFQ

*ALB*：VIC-AAGTGACAGAGTCACCAAATGCTGCACAG-MGBNFQ

**Primers**

*SMN1/SMN2*：

Forward: CTATTTTTTTTAACTTCCTTTATTTTCC

Reverse: GAATGTGAGCACCTTCCTTCTT

*ALB*：

Forward: TGTTGCATGAGAAAACGCCA

Reverse: GTCGCCTGTTCACCAAGGAT
